# Fundamental nursing care in patients with the SARS-CoV-2 virus: results from the ‘COVID-NURSE’ mixed methods survey into nurses’ experiences of missed care and barriers to care

**DOI:** 10.1186/s12912-021-00746-5

**Published:** 2021-11-01

**Authors:** Holly V. R. Sugg, Anne-Marie Russell, Leila M. Morgan, Heather Iles-Smith, David A. Richards, Naomi Morley, Sarah Burnett, Emma J. Cockcroft, Jo Thompson Coon, Susanne Cruickshank, Faye E. Doris, Harriet A. Hunt, Merryn Kent, Philippa A. Logan, Anne Marie Rafferty, Maggie H. Shepherd, Sally J. Singh, Susannah J. Tooze, Rebecca Whear

**Affiliations:** 1 College of Medicine and Health, University of Exeter, St Luke’s Campus, Heavitree Road, Exeter, EX1 2LU UK; 2 School of Health and Society, University of Salford, Allerton Building, Frederick Rd, Salford, M6 6PU UK; 3 Northern Care Alliance NHS Group, Stott Lane, Salford, M6 8HD UK; 4 Department of Health and Caring Sciences, Western Norway University of Applied Sciences, Inndalsveien 28, 5063 Bergen, Norway; 5 The National Institute for Health Research (NIHR) Applied Research Collaboration (ARC) South West Peninsula (PenARC), Exeter, UK; 6 The Royal Marsden NHS Foundation Trust, Fulham Road, London, SW3 6JJ UK; 7 School of Medicine, University of Nottingham, Queens Medical Centre, Nottingham, NG7 2UH UK; 8 Faculty of Nursing, Midwifery and Palliative Care, King’s College London, London, SE1 8WA UK; 9 NIHR Exeter Clinical Research Facility, Royal Devon and Exeter NHS Foundation Trust, Barrack Road, Exeter, EX2 5DW UK; 10 Institute of Biomedical and Clinical Science, College of Medicine and Health, University of Exeter, St Luke’s Campus, Heavitree Road, Exeter, EX1 2LU UK; 11 Department of Respiratory Science, University of Leicester, University Road, Leicester, LE1 7RH UK; 12 University Hospitals of Leicester NHS Trust, Biomedical Research Centre – Respiratory, Glenfield Hospital, Groby Road, Leicester, LE3 9QP UK

**Keywords:** Fundamental nursing care, COVID-19, SARS-CoV-2, Missed care, Survey, Mixed methods

## Abstract

**Background:**

Patient experience of nursing care is associated with safety, care quality, treatment outcomes, costs and service use. Effective nursing care includes meeting patients’ fundamental physical, relational and psychosocial needs, which may be compromised by the challenges of SARS-CoV-2. No evidence-based nursing guidelines exist for patients with SARS-CoV-2. We report work to develop such a guideline. Our aim was to identify views and experiences of nursing staff on necessary nursing care for inpatients with SARS-CoV-2 (not invasively ventilated) that is omitted or delayed (missed care) and any barriers to this care.

**Methods:**

We conducted an online mixed methods survey structured according to the Fundamentals of Care Framework. We recruited a convenience sample of UK-based nursing staff who had nursed inpatients with SARS-CoV-2 not invasively ventilated. We asked respondents to rate how well they were able to meet the needs of SARS-CoV-2 patients, compared to non-SARS-CoV-2 patients, in 15 care categories; select from a list of barriers to care; and describe examples of missed care and barriers to care. We analysed quantitative data descriptively and qualitative data using Framework Analysis, integrating data in side-by-side comparison tables.

**Results:**

Of 1062 respondents, the majority rated mobility, talking and listening, non-verbal communication, communicating with significant others, and emotional wellbeing as worse for patients with SARS-CoV-2. Eight barriers were ranked within the top five in at least one of the three care areas. These were (in rank order): wearing Personal Protective Equipment, the severity of patients’ conditions, inability to take items in and out of isolation rooms without donning and doffing Personal Protective Equipment, lack of time to spend with patients, lack of presence from specialised services e.g. physiotherapists, lack of knowledge about SARS-CoV-2, insufficient stock, and reluctance to spend time with patients for fear of catching SARS-CoV-2.

**Conclusions:**

Our respondents identified nursing care areas likely to be missed for patients with SARS-CoV-2, and barriers to delivering care. We are currently evaluating a guideline of nursing strategies to address these barriers, which are unlikely to be exclusive to this pandemic or the environments represented by our respondents. Our results should, therefore, be incorporated into global pandemic planning.

**Supplementary Information:**

The online version contains supplementary material available at 10.1186/s12912-021-00746-5.

## Background

Patient experience of care is associated with safety, clinical effectiveness, care quality, treatment outcomes, costs and service use [1–5], and nursing care is a key determinant of this experience [6, 7]. Although nurses perform both generalist and specialist roles, all nurses are involved in meeting patients’ ‘fundamental’ care needs. Defining fundamental care has in the past been a contested area [8], but there is now greater consensus [9] in that fundamental care can be described as ‘actions on the part of the nurse that respect and focus on a person’s essential needs to ensure their physical and psychosocial wellbeing’ ( [9], p.2292). These needs are met by developing a positive and trusting relationship with the person being cared for as well as their family/carers [10]. Therefore, the discrete elements of fundamental care can be described as: actions to meet patients’ physical needs, and their psychosocial (wellbeing and mental health) needs; these actions include nurses’ transactional and relational behaviours [9].

The combination of SARS-CoV-2 symptoms and infectiousness of the SARS-CoV-2 virus may pose significant challenges for meeting patients’ physical and psychosocial needs, as well as impacting on nurses’ relational and transactional care behaviours. Such challenges may result in ‘missed care’ or ‘care left undone’, defined as any aspect of nursing care that is omitted or delayed, in part or in whole [11, 12]. Whilst our current study focuses specifically on fundamental nursing care, missed care can therefore include areas of fundamental nursing care (e.g. meeting patients’ hygiene needs) as well as other areas of nursing care (e.g. discharge planning) [13]. Prior to the SARS-CoV-2 pandemic, both nurses and patients indicated that important elements of fundamental nursing care are regularly missed, including nutrition, hygiene (e.g. bathing; mouth care), ambulation/ supporting mobility, communication/ talking with patients, and emotional and psychological support [13–17]. The extent of missed care is related to poor patient outcomes, increased mortality and adverse events, and poor patient satisfaction and experience of care [15, 18–20]. Factors contributing to missed care include high patient to registered nurse ratios, associated lack of nurse time, patient dependency/ acuity, and the practice environment (e.g. managerial support) [12, 16, 17, 21], and the likely impact of the SARS-CoV-2 pandemic on all of these factors may further increase incidences of missed care. Indeed, we know from nurses’ narrative accounts of the Canadian SARS outbreak in 2003 that Personal Protective Equipment (PPE) [22], time pressures [23] and visitor restrictions [24] can lead to patients feeling abandoned by nurses [23].

Given the short time since the SARS-CoV-2 virus first emerged in late 2019, knowledge of its impact on nursing care has been slow to emerge and as yet there is no direct data on patients’ experience of nursing care. Most of the research published to date has focussed on the impact on nurses’ wellbeing, not on their processes of caring. Thus, we know from surveys conducted in China and Italy that front-line nurses have experienced huge workload; long-term fatigue; infection threat; and anxieties and frustration concerning the death of patients for whom they cared. Additionally, they worried about their families and vice versa [25]. A survey completed by 764 nurses (60.8%) and 493 physicians (39.2%) from 34 hospitals in China reported symptoms of depression (634 [50.4%]), anxiety (560 [44.6%]), insomnia (427 [34.0%]), and distress (899 [71.5%]) amongst the health care workers. Nurses, frontline health care workers, female health care workers, and those working in Wuhan, reported more severe mental health symptoms than other health care workers [26]. Nurses in Italy reported Post-Traumatic Stress Disorder symptoms, severe depression, anxiety, insomnia and perceived stress [27], and UK nurses were concerned about the risks of SARS-CoV-2 on their physical and mental health, as well as the health of their families [28, 29].

There is evidence emerging from the UK that these effects on nurses, together with the rapid adjustment of healthcare systems to accommodate the pressures of increased patient flow, redeployment of inexperienced staff into isolation care environments, and other factors, may have had a negative impact on patients. One survey on recovered patients’ experiences at discharge found that 82% of respondents had not received post-discharge assessment visits, and 18% reported having unmet needs [30]. Thus, the negative impact of the SARS-CoV-2 outbreak on nurses may in turn impact patients, acting as a mediator of missed care. However, we do not currently know if the concerns raised in the brief reports from the Canadian SARS outbreak have been replicated or learnt from in this current pandemic, and there are currently no evidence-based guidelines for nursing patients with SARS-CoV-2 who may be receiving ventilatory support without tracheal intubation (i.e. not invasively ventilated), who represent the majority of hospitalised patients with this condition. This leaves nurses without guidance and is potentially associated with variations in patient experience, care quality and costs as redeployed and/or inexperienced nurses struggle to adapt to caring for patients in strict isolation [31] using unfamiliar care procedures required for infection prevention and control [32].

We designed the COVID-NURSE cluster randomised controlled trial [33] to evaluate a fundamental nursing care protocol for patients hospitalised with the SARS-CoV-2 virus not invasively ventilated. The fundamental nursing care protocol has been developed from three main areas of activity: i) a survey of registered nurses’ and non-registered auxiliary nursing/ healthcare support workers and assistants’ views and experiences of caring for patients with SARS-CoV-2 including the barriers encountered in delivering fundamental care and strategies adopted to overcome these; ii) a rapid systematic review of the literature [34]; and iii) four co-creation workshops involving nurses and patients with experience of being hospitalised with the SARS-CoV-2 virus not invasively ventilated, in which the findings from the survey and the systematic review were presented and discussed.

In this paper, we report the findings from the survey of nurses that relate to their views and experiences of missed fundamental care and barriers to fundamental care for inpatients with SARS-CoV-2 not invasively ventilated. We will report findings on strategies used to overcome the barriers to care in a future article, to avoid potential contamination of the trial before completion.

## Methods

### Aim and design

Our aim was to identify the views and experiences of registered nurses and non-registered nursing care staff on missed fundamental care and the barriers to fundamental care for inpatients with the SARS-CoV-2 virus not invasively ventilated. We conducted a cross-sectional study employing a mixed methods explanatory design [35] guided by a pragmatic philosophy [36]. For the quantitative and qualitative components, we collected data concurrently and analysed data sequentially (with qualitative data analysed in order to explain the quantitative data). We gave the quantitative component greater priority as this guided our analysis of qualitative data, and we mixed the components during data analysis. This approach enabled us to utilise the qualitative data to elaborate on, clarify, illustrate and contextualise the key quantitative findings [37, 38].

### Participants and recruitment

Eligible respondents were UK-based registered nurses and non-registered auxiliary nursing/ healthcare support workers/ assistants that had actively engaged in nursing inpatients with the SARS-CoV-2 virus who were not invasively ventilated. Thus, respondents who had nursed patients who were not ventilated, or patients who received non-invasive ventilation, were eligible; respondents who only nursed invasively ventilated patients were ineligible. We invited a convenience sample of respondents using a range of strategies; including a database of nurses who had consented to be approached for SARS-CoV-2 related research studies through their involvement in the ‘Impact of COVID-19 on the Nursing and midwifery workforce’ (ICON) study [29]; networks of senior research, management and clinical nurses in England and Wales including contacts within the National Institute for Health Research (NIHR) 70@70 research network, the Association of UK Lead Research Nurses, the Royal Colleges, and hospital sites affiliated with the COVID-NURSE Trial co-investigators; the UK NIHR Clinical Research Network; and through social media including Twitter and University of Exeter channels. We aimed for our survey distribution channels to be as inclusive as possible in terms of encouraging responses from nurses working in different types of hospitals, including general vs. specialist, which were geographically diverse and serving populations of different ethnicities.

We sent a link to the survey to nurses on our database and to key gatekeepers in the networks listed above. We asked gatekeepers to circulate the link via newsletters, emails and other communication channels appropriate to their networks with a covering letter informing potential respondents of the purpose and timeframe for the survey. The landing page for the survey provided links to the participant information sheet, frequently asked questions, and the survey. As this was an exploratory study to guide our intervention development, within the specific time limits of the COVID-NURSE trial and thus using a convenience sampling frame, we did not have a predetermined sample size calculation and sought to recruit as many respondents as possible during the timeframe of the survey.

### Data collection and materials

The survey was open for 3 weeks, plus 3 days to respondents who had commenced the survey, to provide the opportunity to complete it. We developed the survey with input from the COVID-NURSE trial Co-Investigators and members of the wider research team, in response to formal and informal feedback from four nursing teams who piloted the survey, and in line with comments from the University of Exeter Medical School Ethics Committee. At all times in the development of the survey we involved members of our patient and public involvement group (PPI) including our PPI co-investigators and trial management group member, who gave us advice on the survey design. Using Qualtrics™ online survey software [39], we designed a bespoke online series of survey questions including demographic items. We structured our survey according to the Fundamentals of Care model [8, 9]. We included three sections on physical, relational and psychosocial areas of care, and subsections in each of these areas corresponding to sub-categories of care as adapted from Feo et al. (2018) [9] (Table 1).

**Table 1:** Survey structure; fundamental care areas and sub-categories of care

Section: Care area	Subsection: Sub-category of care
1. Physical	1. Hygiene, personal cleansing and toileting
	2. Eating and drinking
	3. Rest and sleep
	4. Mobility
	5. Patient comfort
	6. Patient safety
	7. Medication management
2. Relational	1. Establishing a relationship with patients
	2. Talking and listening
	3. Non-verbal communication
	4. Shared decision-making
	5. Communicating with relatives, carers and significant others
3. Psychosocial	1. Dignity and respect
	2. Respecting patients’ values and beliefs
	3. Wellbeing, anxiety and depression

In each subsection (Table 1), we asked respondents to:

- Rate how well they thought they were able to meet the needs of patients with the SARS-CoV-2 virus (excluding those who had been invasively ventilated) compared to their ability to meet the needs of other patients they were experienced in nursing before SARS-CoV-2. For example, for section one (physical care), subsection one, respondents were asked to rate how well they were able to meet the hygiene, personal cleansing and toileting needs of patients with the SARS-CoV-2 virus (excluding those who had been invasively ventilated) compared to their ability to meet the hygiene, personal cleansing and toileting needs of other patients they were experienced in nursing before SARS-CoV-2. Respondents were asked to provide answers on a five point Likert-type scale with the following options: much better than, a little bit better than, the same as, a bit worse than, or much worse than. Alternatively, respondents could indicate that they were not involved in this area of care;
- Provide free text to narratively identify and describe examples of missed fundamental care;
- Select all relevant barriers to fundamental care from a list provided. The barrier list was derived from discussions with the COVID-NURSE trial Co-Investigators with expertise in nursing. Barriers were standardised across all sub-categories, with additional barriers added where relevant for a specific category. A list of barriers available for each sub-category is provided in Additional file 1;
- Provide free text to narratively identify and describe examples of barriers to fundamental care.

### Data analysis

The UK Clinical Research Collaboration (UKCRC)-registered University of Exeter Clinical Trials Unit received, cleaned and processed the data, and uploaded datasets to Microsoft Excel [40]. We applied pairwise deletion to each survey item in order to maximise the data available, and reported all percentages as the percentage of the total number of respondents who provided data for that survey item. We combined ethnicity data into standard categories [41].

#### Quantitative data analysis

We analysed quantitative data, including demographic data, descriptively. For respondents’ ratings of care, we combined responses into four categories for ease of interpretation: 1) better than other patients (combining ‘much better’ and ‘a little bit better’); 2) the same as other patients; 3) worse than other patients (combining ‘much worse’ and ‘a bit worse’); 4) not involved in this area of care. For both ratings of care and barriers to care, we calculated the frequency, and percentage, of respondents selecting each option for each sub-category of care. We also calculated the percentage of respondents selecting each barrier in total across each of the three care areas (physical; relational; psychosocial).

#### Qualitative and mixed methods data analysis

We achieved familiarisation with the data through reading survey responses and analysed data using Framework Analysis [42] to allow for both inductive and deductive approaches in combining our study aims/ survey questions with participants’ original accounts [42, 43]. In undertaking qualitative analysis within our explanatory mixed methods design, we focused on explaining the key quantitative findings rather than completing a full, independent analysis of qualitative themes, so that we could understand respondents’ meanings in providing their quantitative responses and focus in on qualitative examples in key problem areas indicated by the quantitative data.

Data interpretation and the development of analytic categories were discussed by a multidisciplinary team trained in qualitative data analysis and consisting of five researchers (HVRS, A-MR, HI-S, DAR, NM) with backgrounds in nursing (3), nursing education (3), mental health services research (2) and clinical research (2). The team was led by HVRS; A-MR, HI-S, NM and HVRS independently coded subsets of the raw data; HVRS double-coded and verified subsets of the data coded by A-MR, HI-S and NM.

For missed care and barriers, we separately coded responses into a framework structured according to the Fundamentals of Care Framework. Within each sub-category of care, we analysed survey responses thematically using a constant comparison approach, and examined similarities and differences in respondents’ accounts in order to categorise the examples of missed care and barriers for each sub-category [44, 45]. For barriers to care, we then focused our analysis on the main barriers highlighted by the quantitative data, categorising the qualitative findings across all sub-categories of care into each of the top five rated barriers for each of the three care areas (physical; relational; psychosocial). We have integrated the quantitative and qualitative data in side-by-side comparison tables [35] organised by the quantitative data (for missed care, from highest to lowest percentage of respondents rating the sub-category of care as ‘worse’; for barriers to care, from highest to lowest percentage of respondents selecting the barrier). In these tables we have included summaries of the qualitative findings and quotes to illuminate these.

We describe our study in line with cross-sectional study reporting guidelines (see Additional file 2 for completed STROBE checklist) [46].

## Results

### Respondent characteristics

From 3rd to 26th August 2020, 1062 eligible respondents consented to provide survey data; 84 of these provided no further data. The number of respondents providing data for each survey item is provided in Additional file 1. Respondent characteristics are summarised in Table 2.

**Table 2:** Respondent characteristics

		N (%)
**Gender**	Female	858 (87.7)
	Male	112 (11.5)
	Prefer not to say	8 (0.8)
**Age**	< 25	98 (10.0)
	26–30	173 (17.7)
	31–40	257 (26.3)
	41–50	234 (23.9)
	51–60	182 (18.6)
	61–66	26 (2.7)
	> 67	1 (0.1)
	Prefer not to say	7 (0.7)
**Ethnicity**	Asian/ Asian British	32 (3.3)
	Black/ African/ Caribbean/ Black British	15 (1.5)
	Mixed/ Multiple ethnic groups	13 (1.3)
	Other ethnic group	46 (4.7)
	Other White	85 (8.7)
	White British	779 (79.7)
	Prefer not to say	8 (0.8)
**Environment**	Acute General NHS hospital including teaching hospital	898 (91.8)
	Tertiary/ specialist	63 (6.4)
	Private healthcare	6 (0.6)
	Missing data	11 (1.1)
**Country**	England	933 (95.4)
	Wales	15 (1.5)
	Scotland	5 (0.5)
	Northern Ireland	4 (0.4)
	Other country	1 (0.1)
	Missing data	20 (2.0)
**Main position**	Charge nurse	206 (21.1)
	Staff nurse	374 (38.2)
	Specialist/ advanced nurse	142 (14.5)
	Research nurse	42 (4.3)
	Nurse researcher	1 (0.1)
	Manager	73 (7.5)
	Student nurse	20 (2.0)
	Non-registered nursing associate	10 (1.0)
	Non-registered care or nursing assistant	90 (9.2)
	Missing data	20 (2.0)
**Redeployed?**	Yes	139 (14.2)
	No	227 (23.2)
	Missing data	612 (62.6)
**Usually work on respiratory ward**^a^**?**	Yes	114 (11.7)
	No	252 (25.8)
	Missing data	612 (62.6)
**Usually work in non-ward**^a^ **environment?**	Yes	138 (14.1)
	No	228 (23.3)
	Missing data	612 (62.6)

^a^Ward: an inpatient division in a hospital typically shared by patients who need a similar type of care. Non-ward: a non-residential health care setting such as outpatient, community or primary care. Percentages may not always total 100 due to rounding

### Respondents’ views on missed fundamental care

#### Quantitative results

**Fig. 1:**
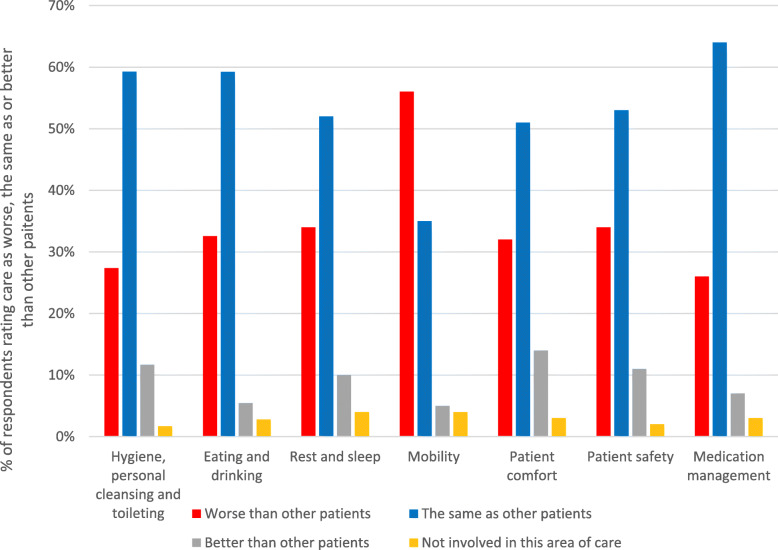
Respondents’ ratings of meeting the physical care needs of patients with SARS-CoV-2

**Fig. 2:**
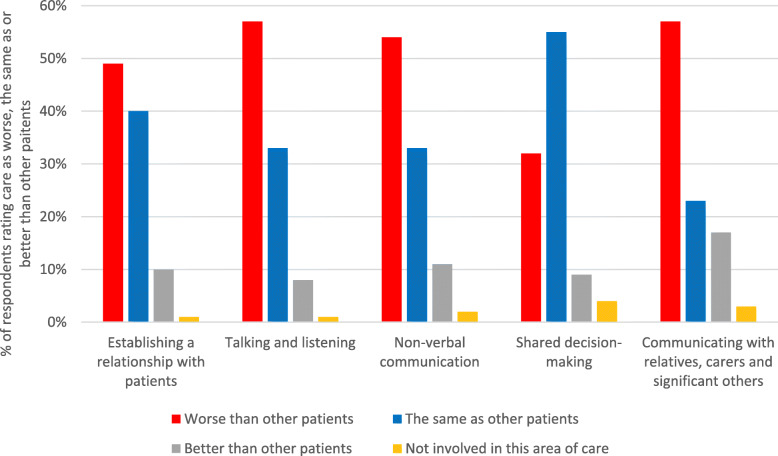
Respondents’ ratings of meeting the relational care needs of patients with SARS-CoV-2

**Fig. 3:**
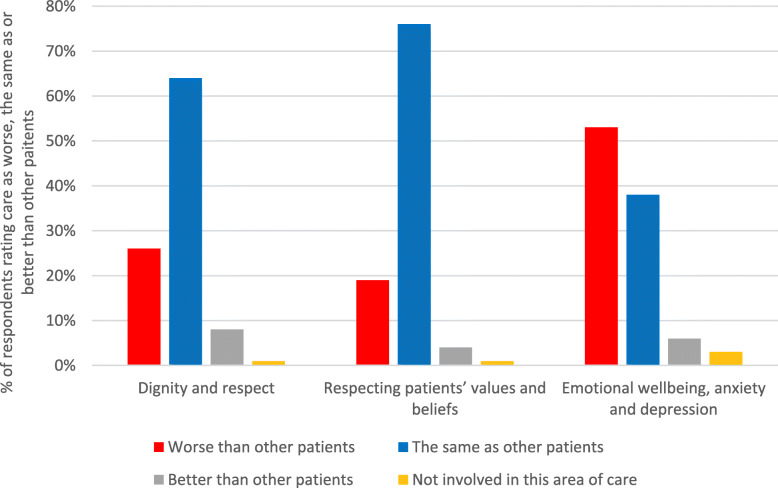
Respondents’ ratings of meeting the psychosocial care needs of patients with SARS-CoV-2

The percentage of respondents rating the physical, relational and psychosocial care of patients with the SARS-CoV-2 as worse, the same as, or better than other patients, for each constituent sub-category of care, is shown in Figs. 1, 2 and 3. Frequencies are provided in Additional file 1.

For sub-categories of care across all three care areas (physical, relational, psychosocial), a majority of respondents rated their ability to meet the needs of SARS-CoV-2 patients as worse than for other patients. The following sub-categories were rated as worse by a majority of respondents: ‘talking and listening’ (57%); ‘communicating with relatives, carers and significant others’ (57%); ‘mobility’ (56%); ‘non-verbal communication’ (54%); ‘emotional wellbeing, anxiety and depression’ (53%). Just less than half (49%) of respondents also rated ‘establishing a relationship with patients’ as worse for SARS-CoV-2 patients. For all the other physical sub-categories (aside from ‘mobility’), approximately one third or less of respondents rated care as worse for SARS-CoV-2 patients (range 26–34%). For relational care, almost one third of respondents (32%) rated ‘shared decision-making’ as worse for SARS-CoV-2 patients, and for psychosocial care ‘dignity and respect’ and ‘respecting patients’ values and beliefs’ were rated as worse for SARS-CoV-2 patients by 26 and 19% of respondents respectively.

#### Integrated quantitative and qualitative results

In Tables 3, 4 and 5 we have presented side-by-side comparison tables integrating the quantitative findings and summarised qualitative findings on missed care for each sub-category of physical, relational and psychosocial care. Respondents’ ID numbers are included in brackets after quotes.

**Table 3:** Side-by-side comparison of quantitative and qualitative data on missed physical care

Care category (% rating care as worse)	Summary of qualitative data on missed care	Quotes demonstrating qualitative data
Mobility (56%)	Patients in side rooms^a^ were unable to mobilise by moving freely on the ward and walking to the toilet. Patients were also unable to access facilities such as the gym or garden, or complete stair assessments. Pressure area care and rehabilitation could be missed, and respondents experienced a lack of physiotherapy presence/ support.	“Due to needing to isolate … one of our patients was in his room for 4 weeks.” (ID581) “Physios would leave C19+ patients to be seen at the end of the day, resulting in a shortened session or missed session.” (ID244)
Rest and sleep (34%)	Patients often experienced sleep which was interrupted by consistent monitoring/ observations and interventions, and by noise and lights. Patients also experienced a lack of time for sleep, difficulties settling, and poor quality sleep.	“There was not much “down time” during the night.” (ID585) “It was so busy that we couldn’t even switch off the lights … sleep deprivation was present on every night shift.” (ID355)
Patient safety (34%)	Respondents highlighted a reduction in monitoring/ observing patients who were in closed bays or side rooms, and a related increased risk of falls. Respondents also noted various errors and potential for errors, such as medication errors and failures to escalate, and that regular skin checks could be missed.	“Need to isolate patients into side rooms was treated (rightly or wrongly) as more important than their risk of falls.” (ID286) “Medication rounds not done next to patient. Nursing staff not present in the bays so often.” (ID512)
Eating and drinking (33%)	Respondents noted issues with food/drink supplies such as running out of drinks, lack of choice for patients, and difficulties for patients eating using plastic plates/cutlery. There were some delays in providing food and drink to patients, and between meals these needs could be missed. Some patients were weighed less regularly and some respondents experienced less presence from dieticians.	“Poor nutritional intake for those most vulnerable. Missed regular cups of tea … Gut-wrenching as a nurse.” (ID20) “Tea trolleys and meal choices were much harder to facilitate. It took longer for diet and fluid to get to patients.” (ID262
Patient comfort (32%)	Symptom control was challenging, particularly breathlessness and temperature, and oxygen equipment was uncomfortable. Patients missed having visitors, and interaction/ time with nurses, which affected their comfort levels. Respondents also noted that nursing patients in the prone position (‘proning’) and difficulties turning patients meant pressure area care could suffer.	“Patients were uncomfortable due to nature of condition, positioning (extended time prone giving them sore joints or back).” (ID475) “Unable to comfort emotionally distressed patients due to PPE. Lack of family support.” (ID464)
Hygiene, personal cleansing and toileting (27%)	Patients’ personal care could be missed or delayed, particularly mouth care, but also washes, hair brushing and toileting. Less time was spent on personal hygiene, with some patients expected to meet these needs themselves yet not encouraged to do so. Patients’ rooms were also cleaned less often, and patients often couldn’t access private bathrooms.	“Delayed response to washes, often flowing into afternoon. Personal care, oral care, often missed completely.” (ID20) “Less assistance. Patient left or expected to meet most hygiene needs themselves and not pushed or encouraged to do this.” (ID130)
Medication management (26%)	Some staff were unable to double check medications and some reported an increase in medication errors. There could be delays in receiving and administering medications, and some respondents ran out of medications. Respondents also experienced a lack of pharmacist presence/support.	“I made my first medication error during the pandemic... Thankfully no one was hurt, but it still haunts me.” (ID529) “Medications were not available … Pharmacists refused to come to the wards.” (ID703)

^a^Side rooms: rooms in which individual patients stay in isolation from other patients

**Table 4:** Side-by-side comparison of quantitative and qualitative data on missed relational care

Care category (% rating care as worse)	Summary of qualitative data on missed care	Quotes demonstrating qualitative data
Talking and listening (57%)	Many respondents highlighted a lack of rapport building with patients, and lack of clear communication with patients (i.e. being heard and understood), specifically noting the inability to lip read through PPE. Related to this, respondents stressed reductions in nurse-patient contact, including both physical touch and time spent with patients.	“Wearing PPE especially masks meant that patients often could not hear you and you would have to repeatedly talk to them which made conversation and flow more difficult.” (ID328) “You can’t hear properly with shields. You can’t see properly. I lip read as well as listen, this is very difficult. Verbal [communication] is difficult with softly spoken patients.” (ID505)
Communicating with relatives, carers and significant others (57%)	Patients missed having visits from significant others and were accordingly isolated. Staff had less opportunity to build relationships with significant others and manage their emotional needs, and noted that significant others were not always updated in a timely and regular manner. Staff experienced difficulties keeping significant others fully informed over the phone whilst ensuring confidentiality was maintained.	“Patients struggled with lack of family contact.” (ID573) “Regular updates with relatives and carers were not always achieved.” (ID427) “It has also been difficult building a rapport with families and relatives... This has added difficulty as they are unable to see the environment their loved one is being care in.” (ID579)
Non-verbal communication (54%)	Staff were less able to communicate with patients using facial expressions, non-verbal cues, physical gestures and touch. Some respondents therefore felt that they were showing less comfort, reassurance, empathy and friendliness towards patients. They also had a reduced ability to pick up on patients’ non-verbal cues and respond to their needs accordingly.	“Patients unable to read facial expressions, see non-verbal cues, see the nurse was being empathetic and compassionate to their needs as they were unable to see nurse’s faces.” (ID332) “Visitors (who normally pick up on missed cues) unable to visit.” (ID204)
Establishing a relationship with patients (49%)	Some respondents highlighted the same issues as experienced in relation to ‘talking and listening’, also noting that it was harder to get to know these patients; that functional care could be prioritised over relationship building; and that they missed opportunities to obtain information about patients from their significant others as usual.	“The human aspect of nursing care. Not being able to smile. To sit and make a cup of tea and listen to the patient’s opinion of how their stay was going. Every aspect of nursing became clinical.” (ID20) “Difficult to hear and communicate whilst wearing PPE, therefore loss of personal touch.” (ID585)
Shared decision-making (32%)	Some respondents experienced decision-making as more rushed and policy-led (e.g. escalation/ resuscitation plans) with somewhat less involvement from the patient and significant others. Staff were also less equipped than normal with the knowledge required to answer patients’ questions and provide information during decision-making.	“Due to nature of virus decisions were often made in patients’ best interests, without being able to discuss them with patient or family.” (ID204) “It was decided that all patients over a certain age would be DNAR, it was hard to justify this.” (ID368)

*DNAR* Do Not Attempt Resuscitation

**Table 5:** Side-by-side comparison of quantitative and qualitative data on missed psychosocial care

Care category (% rating care as worse)	Summary of qualitative data on missed care	Quotes demonstrating qualitative data
Emotional wellbeing, anxiety and depression (53%)	Respondents noted that patients’ physical care was prioritised over their emotional needs. Staff were unable to provide normal levels of support e.g. skin to skin touch; time for communication and listening. Patients experienced isolation; and little interaction with staff, significant others, or other patients. Respondents observed fear and low mood across patients, and were unable to reassure patients with knowledge about the virus/ treatments. Respondents also noted a lack of presence/support from psychological services.	“Some days it was just task orientated and we just needed to get to the end of the shift without anyone dying.” (ID593) “We weren’t able to even give a patient a hand to hold that didn’t have a glove on it and a face covered in a mask.” (ID354) “Unable to refer patients to psychology for support or to have relatives to visit or for patients to go outside.” (ID196)
Dignity and respect (26%)	Some respondents reported experiencing difficulties maintaining privacy and dignity for patients, such as closing curtains when performing personal care. Patients who would normally use the bathroom had to use the commode, and patients had to wear hospital gowns rather than their own clothes. Proning patients was also considered undignified, and some respondents experienced patients dying in bays with no privacy.	“A lack of space, and having often 2 patients in one bedspace meant that privacy was difficult. We had access to some privacy screens, but nowhere near enough.” (ID377) “Proning can be undignified due to the nature of the positioning and amount of people it requires to undertake.” (ID585) “Patients dying next to them was very distressing.” (ID298)
Respecting patients’ values and beliefs (19%)	Some respondents noted a lack of knowledge of patients’ beliefs as patients were unable to inform them and/or significant others were not present to guide them. Respondents experienced a lack of chaplaincy support on the wards. Patients were also unable to leave their rooms to visit the prayer room/ chapel, and could not access family/community support as they normally would.	“We didn’t have chaplaincy visiting. We weren’t able to spend time with our dying patients in the same way. We didn’t always know the patient’s spiritual or religious beliefs. We often didn’t know much at all about them.” (ID354) “Patients from certain cultures were unable to behave in the usual way due to restrictions.” (ID487)

Between 26 and 56% of respondents struggled to meet all sub-categories of patients’ physical needs efficiently and effectively compared to patients they would normally care for. Particularly highlighted were restrictions on patients’ mobilisation outside of side (isolation) rooms, and some respondents also described issues with interrupted sleep; missed and delayed personal care, particularly mouth care; restrictions and delays in providing food and drink; reductions in observing patients in side rooms; errors and potential for errors, particularly around medications; challenges with controlling symptoms of breathlessness and high temperature; missed pressure area care; and a lack of presence from multidisciplinary colleagues such as physiotherapists, dieticians and pharmacists.

In relational care, the majority of respondents (57%) highlighted communication difficulties with patients and their significant others, with almost half of respondents reporting that this impacted on their ability to establish a relationship with patients. Respondents struggled to build rapport with patients; experienced restrictions in being heard, understood, and spending time with patients; and were less able to use facial expressions, non-verbal cues and touch to comfort patients. Respondents reported that patients missed having visits from significant others, and they struggled to both keep significant others informed and obtain information about patients from them. A third of respondents also experienced shared decision-making as more rushed and policy-led than usual.

In psychosocial care, the majority of respondents (53%) reported struggling to support patients’ emotional wellbeing and mental health, typically prioritising functional and physical care over emotional care. Most respondents were unable to provide usual levels of support, reassurance and interaction with patients, and reported that patients experienced isolation, loneliness, fear and low mood. A minority of respondents also had difficulties maintaining patients’ privacy and dignity, such as drawing curtains for personal care or distressing scenes; lacked knowledge about patients’ beliefs; and noted a lack of presence from psychological services and chaplaincy.

### Respondents’ views on barriers to fundamental care

#### Quantitative results

We summarise the percentage of respondents selecting each barrier in Table 6 (presented as the average percentage of respondents selecting the barrier, and the range of respondent percentages, across the constituent sub-categories of physical, relational and psychosocial care; where the barrier was only an option for one sub-category, we just present the percentage of respondents selecting the barrier for that sub-category). We have listed barriers in the order of most to least frequently selected across all care areas. We have provided the number of respondents selecting each barrier for each sub-category of care in Additional file 1.

**Table 6:** Percentage of respondents selecting each barrier to care (average and range across sub-categories)

Barrier	Physical Care	Relational Care	Psychosocial care
Wearing PPE	33% (21–44%)	61% (37–77%)	44% (36–48%)
Severity of the patient’s condition	41% (33–50%)	29% (21–47%)	37% (35–39%)
Difficulties taking items / equipment in and out of isolation rooms for patients nursed in these environments	38% (28–54%)	13% (9–18%)	17% (13–21%)
Lack of time	22% (12–29%)	31% (24–41%)	37% (30–40%)
Lack of personnel, skill mix, catering, housekeeping or dietetic support	30% (11–38%)	12% (9–17%)	24% (20–27%)
Lack of knowledge about COVID-19	23% (13–34%)	16% (8–28%)	25% (18–30%)
Not enough physical resources such as equipment/washing facilities/stock items e.g. water jugs, disposable cups, patients’ teeth	24% (12–28%)	12% (4–34%)	14% (10–19%)
Fear of catching COVID-19	21% (13–30%)	19% (11–25%)	23% (22–24%)
Frequent changes in hospital, Trust or organizational policies	22% (15–35%)	12% (7–20%)	15% (14–15%)
Lack of appropriate PPE	17% (11–26%)	8% (5–11%)	10% (10%)
Lack of ability to establish a meaningful rapport with the patient	9% (5–17%)	15% (12–22%)	14% (11–19%)
Lack of information about the ward or patient	10% (6–14%)	9% (7–11%)	16% (12–24%)
Competing requirements of essential medical interventions	15% (13–20%)	9% (4–11%)	11% (10–13%)
Lack of ability to regulate the environment (noise level, lighting, remote monitoring)	42% (34–49%)	N/A	N/A
Lack of relevant personal expertise	11% (6–16%)	7% (4–11%)	11% (9–12%)
Lack of personal psychological support	7% (5–10%)	7% (5–10%)	11% (7–17%)
Lack of personal emotional capacity	6% (2–9%)	7% (5–10%)	9% (7–14%)
Lack of access to changing facilities for PPE	9% (6–17%)	4% (2–5%)	5% (4–7%)
Lack of leadership from senior nurses or managers	8% (5–13%)	6% (3–9%)	8% (7–10%)
Lack of privacy for the patient	51%^a^	N/A	N/A
Inability to meet the patient’s dietary requirements	13%^a^	N/A	N/A
Other	5% (3–7%)	5% (2–17%)	7% (6–9%)

^a^No range provided as barrier only offered as an option for one sub-category of care

In total, eight barriers were ranked within the top five in at least one of the three care areas (Tables 7, 8 and 9). ‘Wearing PPE’, and the ‘severity of the patient’s condition’ were the most frequently selected barriers and were among the top five barriers to all three care areas. The third most frequently selected barrier, ‘difficulties taking items/ equipment in and out of isolation rooms’, was among the top five barriers to physical care. ‘Lack of time’, the fourth most frequently selected barrier, was among the top five barriers to both psychosocial and relational care. ‘Lack of personnel, skill mix, catering, housekeeping or dietetic support’, the fifth most frequently selected barrier, was among the top five barriers to both physical and psychosocial care. The sixth most frequently selected barrier, ‘lack of knowledge about COVID-19’, was among the top five barriers to both relational and psychosocial care. The seventh most frequently selected barrier, ‘not enough physical resources such as equipment/ washing facilities/ stock items’, was the final top five barrier to physical care. The eighth most frequently selected barrier, ‘fear of catching COVID-19’, was the final top five barrier to relational care.

**Table 7:** Integration of quantitative and qualitative data on top five barriers to physical care

Barrier (% selecting barrier)	Highest sub-category (% selecting barrier)^a^	Experiences/ explanations of barriers (from qualitative data)	Quotes demonstrating qualitative data
Severity of the patient’s condition (41%)	Rest and sleep (50%)	Patients were often fatigued, weak, breathless, bedbound, proned, and had high oxygen requirements. These factors caused discomfort, limited patients’ ability to voice their needs, impeded mobility, interfered with eating/drinking, and restricted staffs’ ability to provide personal care (e.g. bathing; mouth care). The need for frequent monitoring also interrupted patients’ rest.	“CPAP hood made eating and drinking opportunities limited … Their rest and sleep was broken to perform essential care.” (ID988) “The priorities had to change due to maintaining organ functions that were in critical states and personal care had to be pushed down the priority list.” (ID92)
Difficulties taking items/ equipment in and out of isolation rooms (38%)	Eating and drinking (54%)	Staff spent extra time cleaning items which had entered patients’ rooms, and struggled to prepare all items ready to take in to avoid donning and doffing PPE. Specific difficulties included providing meals/drinks and equipment to support mobility, removing plates/trays and waste products, and not being able to take drug charts into rooms.	“There was a big time lag of having to don and doff in and out of rooms if you forgot a flush, or needed another syringe.” (ID20) “The rooms for isolated patients were very small and taking equipment in and out and cleaning equipment … was very time consuming.” (ID456)
Wearing PPE (33%)	Hygiene, personal cleansing and toileting (44%)	Respondents struggled to meet patients’ physical needs, especially personal care and moving/turning patients, whilst wearing PPE which was hot, difficult to see through, and created a physical barrier. Changing PPE between patients took time away from meeting their needs, and donning PPE delayed responding to patients’ requests.	“PPE gear has made delivery of any nursing care so much harder, just by the uncomfortable wearing of the masks, vision obscured by visas or goggles and the heat.” (ID590) “Requirement to wear PPE competes with need to provide assistance promptly.” (ID286)
Lack of personnel, skill mix, catering, housekeeping or dietetic support (30%)	Patient safety (38%)	Respondents noted a lack of physiotherapists, dieticians, pharmacists and domestic staff, which delayed patient care. Nursing staff shortages were stressed, which meant insufficient staff for tasks such as mobilising patients and performing personal care. Redeployed staff could also lack knowledge of equipment, medications and the importance of fundamental care.	“Pharmacists did not visit the ward and they are normally there to support and order drugs so it was another thing that we had to do” (ID83) “We had plenty of redeployed staff but not always staff that were able to be hands on as they had not been clinical for many many years.” (ID179)
Not enough physical resources such as equipment (24%)	Patient comfort (28%)	Respondents reported shortages of items including feed pumps, chairs, hoists, food, hot drinks, bottled water, weighing equipment, curtains, commodes, soap, wipes, medications and PPE. This was related to a lack of storage in COVID-19 areas, inability to share items with other areas, and need to clean items between uses. This impeded patient mobilisation and the timely completion of tasks.	“Chronic shortage of pretty much everything.” (ID80) “Simple things like a tray to take food in to isolation rooms in short supply” (ID98) “Lack of specialist equipment on COVID wards (due to storage or lack of enough equipment to spread between cohort/non-cohort ward)” (ID4)

^a^Highest sub-category = sub-category for which the highest percentage of participants selected the barrier

**Table 8:** Integration of quantitative and qualitative data on top five barriers to relational care

Barrier (% selecting barrier)	Highest sub-category(% selecting barrier)^a^	Experiences/ explanations of barriers (from qualitative data)	Quotes demonstrating qualitative data
Wearing PPE (61%)	Non-verbal communication (77%)	Wearing PPE impeded respondents’ abilities to communicate with patients and build a rapport with them. It was difficult to hear and be heard and understood through PPE; not possible for patients to lip read; and not possible for staff to communicate using non-verbal cues, facial expressions/ smiles, physical gestures or physical touch. PPE also hid staffs’ names, made them harder to recognise, and caused fear for some patients.	“The reduction in the human aspect of nursing, being wrapped in plastic and shouting at people who can’t read your face or mouth was horrible.” (ID20) “It was so difficult to comfort without touch. It was quite alien really.” (ID479) “We would be very scary to patients in the beginning, as they were not used to staff being in full gowns with masks.” (ID39)
Lack of time (31%)	Communicating with relatives, carers and significant others (41%)	Due to staff being off sick, many patients being very unwell, and increased workloads, respondents were often too busy to sit with, talk and listen to patients, and prioritised a functional approach to care. Clustering care, and minimising time spent in patients’ rooms, also meant that staff spent less time with patients. Respondents also lacked time to contact and update patients’ significant others.	“Due to the pressures of the pandemic on staffing and resources sometimes it was hard to spend as much time talking to the patients as would be desired.” (ID618) “Not being able … to sit and make a cup of tea and listen to the patient’s opinion of how their stay was going. Every aspect of nursing became clinical.” (ID20)
Severity of the patient’s condition (29%)	Shared decision-making (47%)	Patients were often sedated, using oxygen equipment or experiencing delirium, which made it difficult for them to communicate, understand their care, be involved in decision-making, and contact significant others. Visitor restrictions due to the nature of COVID-19 meant that staff could not get to know patients through their significant others.	“Patients were sedated so unable to make relationships with them. When they were awake a lot experienced delirium.” (ID851) “There were many times when patients were not well enough and deteriorating so rapidly that we did not really have the time to explain every available option.” (ID377)
Fear of catching COVID-19 (19%)	Establishing a relationship with patients (25%)	Respondents were reluctant to get physically close to patients, and to enter or spend time in their rooms, for fear of contracting COVID-19. They were often advised to minimise time in the patient’s room. Some respondents noted that the quality of PPE did not seem adequate, which led to further fear of contracting COVID-19.	“Peoples’ fear of catching covid meant they rushed time with the patient and didn’t engage with them as much.” (ID558) “Inadequate PPE meant I didn’t want to stay in room for longer than necessary therefore I didn’t spend extra time getting to know the patient.” (ID160)
Lack of knowledge about COVID-19 (16%)	Shared decision-making (28%)	Respondents could lack sufficient knowledge of COVID-19 to provide patients with reassurance and answers about their condition, treatment and likely outcomes as much as they usually would. This impeded building a rapport with patients and shared decision-making.	“[It was] challenging to reassure them and build a rapport with them, particularly when we couldn’t give them much information on the condition or its management (due to general limited knowledge).” (ID465)

^a^Highest sub-category = sub-category for which the highest percentage of participants selected the barrier

**Table 9:** Integration of quantitative and qualitative data on top five barriers to psychosocial care

Barrier (% selecting barrier)	Highest sub-category (% selecting barrier)^a^	Experiences/ explanations of barriers (from qualitative data)	Quotes demonstrating qualitative data
Wearing PPE (44%)	Emotional wellbeing, anxiety and depression (48%)	Wearing PPE limited verbal and non-verbal communication, rapport building, and physical contact with patients, which impacted on patients’ wellbeing and staffs’ abilities to develop therapeutic relationships and meet patients’ emotional needs. Seeing staff in PPE and being unable to recognise them caused discomfort.	“Wearing full PPE impaired the creation of a therapeutic relationship with the patient. Both patient and staff become de-personalised.” (ID443) “It must have been terrifying for the patients who did wake up seeing us in our full PPE.” (ID80)
Lack of time (37%)	Emotional wellbeing, anxiety and depression (40%)	In the context of visitor restrictions, respondents experienced more pressure to provide emotional care to patients, but less time to do so as wards were so busy. Staff had little time to sit with patients, provide support, understand their values and beliefs, and attend to their emotional and spiritual needs. They were also reluctant to spend much time in patient rooms or advised not to.	“In critical care we are used to providing the emotional and psychological support needed, but … Covid critical care being busier than usual caused a lot of constraints to do this.” (ID229) “Lack of time would be the main factor as staff couldn’t fully engage with the patient to understand their beliefs and wishes.” (ID85)
Severity of the patient’s condition (37%)	Dignity and respect (39%)	As many patients were sedated, ventilated and/or short of breath, staff had little opportunity to communicate with them, develop a rapport, assess their emotional/spiritual needs, or find out their wishes and beliefs. Patients and respondents were at times aware when they were likely to die which was overwhelming for all involved.	“The patients were unable to voice their needs.” (ID268) “Patients’ anxieties were difficult to assess at times.” (ID627) “Difficult to establish a rapport with patients as they were so short of breath/wearing CPAP masks, therefore difficult to know what their values/ beliefs are.” (ID585)
Lack of knowledge about COVID-19 (25%)	Emotional wellbeing, anxiety and depression (30%)	Due to their lack of knowledge of COVID-19, respondents found it difficult to answer patients’ questions and reassure them about their care and likely outcomes. Patients ‘feared the unknown’ and had anxiety around the lack of COVID-19 knowledge. Given a lack of knowledge of how COVID-19 may impact on patients psychologically, respondents were reacting to this on an ad hoc basis.	“Because it was so new for us too, sometimes it was difficult to answer their questions.” (ID13) “Lack of knowledge of the disease meant that we were unable to reassure patients about their care and how they were improving.” (ID161) “Patients were very anxious about Covid-19 as so much [is] still unknown.” (ID204)
Lack of personnel, skill mix, catering, housekeeping or dietetic support (24%)	Dignity and respect (27%)	Due to the COVID-19 risk and some PPE scarcity, some respondents reported that psychology reviews were delayed or not undertaken, and chaplaincy/ religious persons were unavailable, even in end of life scenarios. General staff also had a lack of experience and expertise for identifying and supporting patients with psychological problems.	“As no one could come onto the wards we were unable to get a priest or Imam to come and give religious support.” (ID37) “Lack of experience and expertise in general staff for identifying psychological problems and helping patients deal with emotional consequences of illness.” (ID4)

^a^Highest sub-category = sub-category for which the highest percentage of participants selected the barrier

#### Integrated quantitative and qualitative results

In Tables 7, 8 and 9 we report the top five barriers per care area in order of highest to lowest percentage of respondents selecting this barrier across the constituent sub-categories. We also include the sub-category for which the highest percentage of respondents selected the barrier in that care area, and then integrate the qualitative data in side-by-side comparison tables. Respondents’ ID numbers are included in brackets after quotes.

Respondents struggled to meet the physical care needs (particularly rest and sleep, mobility, eating/drinking, and personal care) of patients who were very unwell, often attached to oxygen equipment, and with high monitoring requirements. Wearing PPE also created a physical barrier to meeting such needs, particularly hygiene needs. A lack of storage and stock supplies in SARS-CoV-2 areas, and the increased need to clean items between uses, meant insufficient supplies for respondents to meet patients’ needs and make them comfortable. The need to don and doff PPE upon entering and leaving a patient’s room delayed responding to patients’ needs, meant staff had to try to prepare all items needed in the room before entering, and created difficulties providing patients with items such as food and drinks. Respondents also experienced staff shortages and a lack of presence from specialised services which increased their own workloads, and compromised patients’ care and safety.

Wearing PPE created a very significant barrier to relational care and communicating with patients, compromising hearing, lip reading, seeing facial expressions, use of non-verbal cues and touch. PPE also made staff difficult to recognise. Due to staff shortages and high patient acuity, respondents had little time to spend talking and listening to patients, prioritising a functional approach to care. This was compounded by respondents’ reluctance to spend time in patients’ rooms due to fear of catching SARS-CoV-2, which limited their ability to establish a relationship with patients. Patients’ ability to communicate and participate in decision-making was also compromised by the severity of their own conditions, and respondents felt they lacked the knowledge of SARS-CoV-2 required to answer patients’ questions during the decision-making process in particular. Running through these accounts was the impact of restrictions on visitors, for both patients’ wellbeing and staff’s ability to communicate with significant others, particularly in the context of a lack of time to update significant others remotely and consult on time critical decision-making.

Respondents experienced similar barriers to psychosocial care as to relational care, including the impact of restrictions on visitors. Patients’ wellbeing and staffs’ ability to create therapeutic relationships suffered as a result of the barriers to communication presented by PPE. Again, staff had limited time to spend supporting patients’ emotional and spiritual needs; patients’ abilities to communicate their needs and beliefs were compromised by the severity of their conditions; and staff found it difficult to answer patients’ questions about SARS-CoV-2 or provide them with reassurance. The lack of SARS-CoV-2 knowledge also caused fear and anxiety for patients. Respondents experienced a lack of presence from psychology services and chaplaincy which limited support for patients, and some lacked the expertise to support patients’ psychological needs.

## Discussion

In this survey, we found that respondents rated their ability to meet patients’ needs in many areas of fundamental care as worse for hospitalised patients with SARS-CoV-2 than for other patients they were experienced in caring for before the pandemic. Although meeting patients’ needs was rated by some respondents as worse in all areas of care; the majority of respondents specifically identified mobility; talking and listening; non-verbal communication; communicating with relatives, carers and significant others; and caring for patients’ emotional wellbeing, anxiety and depression as poorer. Across the categories of physical, relational and psychosocial care, we found the strongest reports of difficulties were with undertaking relational care, with the majority of respondents rating their ability to meet patients’ needs as worse in three of the five categories. In contrast, although 26–34% of respondents rated elements of physical care as worse, it was only for mobility that the majority (56%) cited deficits in this care area specifically. For psychosocial care, most respondents thought that their ability to meet patients’ needs was at least as good or better in the areas of respect, dignity, values and beliefs but this contrasts with more than half reporting that their ability to address patients’ emotional wellbeing, depression and anxiety needs was poorer.

Respondents identified clear reasons why they thought these elements of care were worse for these patients. Foremost was infection control, specifically the wearing of PPE and nursing patients in isolation. PPE was cited as a barrier to relational care by twice as many respondents as any other barrier. It was uncomfortable for respondents, created a barrier to providing physical care, and impeded verbal and non-verbal communication. Insufficient stock, and staffs’ inability to take items in and out of isolation rooms without donning and doffing PPE, were also significant barriers to physical care. These barriers were compounded by the severity of the patient’s condition, requiring as it did the use of oxygen equipment, proning (nursing patients in the prone position), and causing patients’ difficulties in communicating when breathless and/or sedated. Another top five barrier to physical and psychosocial care was the lack of presence from specialist services, and a lack of expertise in redeployed staff themselves. Time, or the lack of it, prevented respondents from talking and listening to patients, although another highly cited barrier was staffs’ own reluctance to spend time with patients for fear of catching SARS-CoV-2 themselves. This was compounded by a lack of knowledge about SARS-CoV-2 which impeded respondents’ ability to answer patients’ questions. Throughout their accounts, respondents noted the impact of restrictions on visitors for both patients and nursing staff. This series of barriers conspired to mean that nurses largely focused on the functional, physical aspects of care, with relationship-building and addressing patients’ emotional wellbeing becoming a secondary priority.

Our results support previous work in related areas. With previous studies suggesting that fundamental nursing care is already regularly missed in the areas of mobility, communication and talking with patients, and providing emotional and psychological support [13–17], our findings highlight the further impact of the SARS-CoV-2 virus on meeting patients’ needs in these particular areas. Consistent with previous explanations for missed care prior to the pandemic [12, 16, 17, 21], respondents highlighted patient acuity and lack of time as barriers to meeting patients’ needs, amongst other challenges more specific to the pandemic context.

Respondents’ accounts of the impact of wearing PPE on communication and the development of therapeutic relationships with patients, and the impact of restrictions on visitors for patients’ emotional wellbeing at a time when nurses themselves struggled to meet these needs due to time pressures and PPE, reflect nurses’ non-empirical accounts of the Canadian SARS outbreak in 2003 [22–24]. Our findings concur with nurses’ reports of high workloads and their fears regarding the risks posed by SARS-CoV-2 to themselves [25, 28, 29]. Respondents’ accounts regarding the impact of these factors are consistent with the reports of patients themselves, who may experience poor communication, a lack of support and assistance, and insufficient information and/or equipment [30]. In addition, relatives, carers and significant others can experience poor communication from hospital staff and may not be kept well informed about the patient [30]. Indeed, our findings regarding respondents’ lack of knowledge about SARS-CoV-2 for providing patients with information, the barriers they report to communication with both patients and their significant others, and the lack of supplies reported may help to explain such experiences.

### Strengths and limitations

A key strength of this study is that it is the first to focus on fundamental nursing care in the context of SARS-CoV-2. Despite the relationship between fundamental nursing care, patient experience, treatment outcomes and costs, investigation into fundamental nursing care in the heavily compromised environment of SARS-CoV-2 is not an objective of current SARS-CoV-2 research programmes. Prior to this study we lacked knowledge as to the specific impact of the SARS-CoV-2 virus on meeting patients’ fundamental physical, relational and psychosocial needs.

A further strength is our use of a mixed methods explanatory design. By collecting and integrating quantitative and qualitative data, we have produced a more comprehensive, in-depth and insightful portrait of the issues under investigation [35, 36, 47, 48]. To enhance the usefulness of our results for informing a future nursing protocol, we have used our qualitative findings to explain, illustrate and contextualise our quantitative findings, with a pragmatic focus on understanding the key issues which our nursing protocol needs to address [36–38]. However, it is possible that full, independent thematic analysis of the qualitative data may have highlighted different aspects of the data and presented the data in a different light.

A potential limitation of this study is the absence of a predefined sample size. We employed a convenience sampling frame with a sample size determined by the period of time the survey was open for, which was in turn constrained by the rapid nature of the wider COVID-NURSE trial and the need to gather pandemic evidence as quickly as possible in a rapidly evolving situation. We also experienced respondent fatigue whereby the number of respondents providing data generally reduced as they worked through the survey items [49]. However, we were not seeking to generalise our findings beyond the sampled population via inferential statistical methods which would have formed the basis of any sample size calculation [50]. Furthermore, in all qualitative items we did reach data saturation, at which point data from additional respondents was no longer providing additional clarity or insight; thus, our sample size could be considered adequate as well as appropriate (as our eligibility criteria ensured respondents were experts in the area of interest) [51, 52].

It may have been beneficial to include Allied Health Professionals (AHPs) within our sample; however, time constraints and timelines for additional ethical approvals were prohibitive. The inclusion of AHPs may have provided additional insights, particularly in relation to our finding that a lack of presence from AHPs was considered a barrier to fundamental care by respondents. In addition, given the proportions of different staff completing the survey, our results largely represent the views of registered nurses rather than non-registered members of the nursing workforce who may have held differing views or offered additional insights.

### Implications and future research

Whilst our findings concur with non-empirical reports from previous pandemics [22–24], to our knowledge this study is the first to quantify nurses’ own views as to the impact of a highly infectious virus on their work, and to combine this with qualitative insights to help explain why specific elements of fundamental care are affected. As such, it provides clear guidance for educators, clinicians, managers and policy makers on what to expect and prepare for in caring for patients in pandemic situations.

Further research should build on these findings, addressing the following remaining questions: what is the impact of nurses’ self-reports of missed fundamental care on missed fundamental care as experienced by patients?; what are the implications on patient safety issues such as reduced mobility, infection, and malnutrition, which are often related to missed fundamental care?; what strategies can be incorporated in nursing care to reduce the frequency and impact of missed fundamental care? In our case, we have used these findings together with further information from our systematic review, and from nurses and patients themselves on strategies to confront the barriers to fundamental care identified in this study, to devise a guideline and nursing protocol for pandemic situations. We are currently testing this protocol in a cluster randomised controlled trial [33].

Although our data were collected from a specific nursing context – inpatient care for patients with the SARS-CoV-2 virus who were not invasively ventilated – we suggest that our results have the potential for generalising to other care environments and other pandemic situations globally. Most of the concerns and barriers identified by our respondents are not specific to the SARS-COV-2 virus, nor their particular nursing environment. Communication is at the heart of all good nursing care globally, as it establishes the platform for compassionate and collaborative transactional care which addresses patients’ physical and psychosocial needs [8, 9]. Intrinsic too is the organisation of care and inter-professional working, as well as nursing education about the epidemiology of illnesses and on specific techniques to be used. All these areas are not exclusive to the current SARS-CoV-2 pandemic and we suggest, therefore, that our results will be useful for others devising strategies to support nursing care in other environments (such as care homes), other countries and for other pandemics.

## Conclusions

In a survey of nurses caring for patients in hospital with the SARS-COV-2 virus not invasively ventilated, the majority of respondents rated their ability to meet the needs of these patients as worse than for patients they normally care for in five of 15 specific fundamental care areas. These areas included one physical, three relational and one psychosocial nursing care area, highlighting that communication with patients and their significant others was the major and consistent concern for nurses, alongside organising care (especially mobilisation) for patients nursed in isolation, and addressing patients’ emotional wellbeing and mental health. The major barriers to fundamental care were the wearing of PPE, the severity of these patients’ conditions, lack of time, difficulties taking items and equipment in and out of isolation rooms, lack of interdisciplinary input, lack of knowledge about SARS-CoV-2, and fears of catching the illness itself. The difficulties faced by nurses in establishing relationships with patients led to concerns that care became merely functional and not individualised or patient-centred. These concerns are unlikely to be specific to the SARS-CoV-2 pandemic nor the hospital environments represented by our survey respondents. These results should, therefore, be incorporated into subsequent global pandemic planning by nursing leaders.

## Supplementary Information

**Additional file 1.** Number of respondents providing data per survey item/ selecting each option within each survey item.

**Additional file 2.** Completed STROBE 2007 (v4) Statement—Checklist of items that should be included in reports of cross-sectional studies.

## Data Availability

The datasets used and/or analysed during the current study are available from the corresponding author on reasonable request.

## Abbreviations

ARC: Applied Research Collaboration
AHP: Allied Health Professionals
COVID-19: Coronavirus disease 2019
CPAP: Continuous Positive Airway Pressure
DNAR: Do Not Attempt Resuscitation
ICON: Impact of COVID-19 on the Nursing and midwifery workforce
MRC: Medical Research Council
NIHR: National Institute of Health Research
NHS: National Health Service
PenARC: Applied Research Collaboration South West Peninsula
PPE: Personal Protective Equipment
PPI: Patient and Public Involvement
RCN: Royal College of Nursing
SARS-COV-2: Severe acute respiratory syndrome coronavirus 2
STROBE: Strengthening The Reporting of OBservational studies in Epidemiology
UKCRC: United Kingdom Clinical Research Collaboration
