# Supplementary material for: Fundamental nursing care in patients with the SARS-CoV-2 virus: results from the ‘COVID-NURSE’ mixed methods survey into nurses’ experiences of missed care and barriers to care
Source: BMC Nurs. 2021 Nov 1;20:215. doi: 10.1186/s12912-021-00746-5 (PMC8558545; doi:10.1186/s12912-021-00746-5)
Supplement: Supplementary file 1 — Additional file 1. Number of respondents providing data per survey item/ selecting each option within each survey item. [file 12912_2021_746_MOESM1_ESM.docx]

Fundamental nursing care in patients with the SARS-CoV-2 virus: results from the ‘COVID-NURSE’ mixed methods survey into nurses’ experiences of missed care and barriers to care

Holly V. R. Sugg

Anne-Marie Russell

Leila M. Morgan

Heather Iles-Smith

David A. Richards

Naomi Morley

Sarah Burnett

Emma J. Cockcroft

Jo Thompson Coon

Susanne Cruickshank

Faye E. Doris

Harriet A. Hunt

Merryn Kent

Philippa A. Logan

Anne-Marie Rafferty

Maggie H. Shepherd

Sally J. Singh

Susannah J. Tooze

Rebecca Whear

Additional file 1: Number of respondents (% of total respondents) providing data per survey item/ number of respondents selecting each option within each survey item

| **Survey item** | **Option** | **N (%)** |
| --- | --- | --- |
| **Gender** | Total (see table 2 for each option) | 978 (100) |
| **Age** | Total (see table 2 for each option) | 978 (100) |
| **Ethnicity** | Total (see table 2 for each option) | 978 (100) |
| **Environment** | Total (see table 2 for each option) | 967 (98.9) |
| **Country** | Total (see table 2 for each option) | 958 (98.0) |
| **Main position** | Total (see table 2 for each option) | 958 (98.0) |
| **Redeployed?** | Total (see table 2 for each option) | 366 (37.4) |
| **Usually work on respiratory ward?** | Total (see table 2 for each option) | 366 (37.4) |
| **Usually work in non-ward environment?** | Total (see table 2 for each option) | 366 (37.4) |
| **Hygiene, personal cleansing and toileting: ratings of care compared to other patients** | Worse | 225 |
|  | Same | 487 |
|  | Better | 96 |
|  | Not involved | 14 |
|  | Total | 822 (84.0) |
| **Eating and drinking: ratings of care compared to other patients** | Worse | 210 |
|  | Same | 382 |
|  | Better | 35 |
|  | Not involved | 18 |
|  | Total | 645 (66.0) |
| **Rest and sleep: ratings of care compared to other patients** | Worse | 190 |
|  | Same | 292 |
|  | Better | 57 |
|  | Not involved | 21 |
|  | Total | 560 (57.3) |
| **Mobility: ratings of care compared to other patients** | Worse | 297 |
|  | Same | 188 |
|  | Better | 25 |
|  | Not involved | 21 |
|  | Total | 531 (54.3) |
| **Patient comfort: ratings of care compared to other patients** | Worse | 173 |
|  | Same | 277 |
|  | Better | 74 |
|  | Not involved | 16 |
|  | Total | 540 (55.2) |
| **Patient safety: ratings of care compared to other patients** | Worse | 181 |
|  | Same | 281 |
|  | Better | 57 |
|  | Not involved | 9 |
|  | Total | 528 (54.0) |
| **Medication management: ratings of care compared to other patients** | Worse | 119 |
|  | Same | 297 |
|  | Better | 33 |
|  | Not involved | 13 |
|  | Total | 462 (47.2) |
| **Establishing a relationship with patients: ratings of care compared to other patients** | Worse | 236 |
|  | Same | 191 |
|  | Better | 50 |
|  | Not involved | 6 |
|  | Total | 483 (49.4) |
| **Talking and listening: ratings of care compared to other patients** | Worse | 261 |
|  | Same | 151 |
|  | Better | 36 |
|  | Not involved | 6 |
|  | Total | 454 (46.4) |
| **Non-verbal communication: ratings of care compared to other patients** | Worse | 238 |
|  | Same | 144 |
|  | Better | 48 |
|  | Not involved | 8 |
|  | Total | 438 (44.8) |
| **Shared decision-making: ratings of care compared to other patients** | Worse | 129 |
|  | Same | 219 |
|  | Better | 35 |
|  | Not involved | 16 |
|  | Total | 399 (40.8) |
| **Communicating with relatives, carers and significant others: ratings of care compared to other patients** | Worse | 229 |
|  | Same | 91 |
|  | Better | 66 |
|  | Not involved | 13 |
|  | Total | 399 (40.8) |
| **Dignity and respect: ratings of care compared to other patients** | Worse | 104 |
|  | Same | 254 |
|  | Better | 32 |
|  | Not involved | 5 |
|  | Total | 395 (40.4) |
| **Respecting patients’ values and beliefs: ratings of care compared to other patients** | Worse | 75 |
|  | Same | 297 |
|  | Better | 14 |
|  | Not involved | 5 |
|  | Total | 391 (40.0) |
| **Emotional wellbeing, anxiety and depression: ratings of care compared to other patients** | Worse | 202 |
|  | Same | 145 |
|  | Better | 23 |
|  | Not involved | 11 |
|  | Total | 381 (39.0) |
| **Hygiene, personal cleansing and toileting: textual data on missed care** |  | 191 (19.5) |
| **Eating and drinking: textual data on missed care** |  | 189 (19.3) |
| **Rest and sleep: textual data on missed care** |  | 154 (15.7) |
| **Mobility: textual data on missed care** |  | 175 (17.9) |
| **Patient comfort: textual data on missed care** |  | 149 (15.2) |
| **Patient safety: textual data on missed care** |  | 144 (14.7) |
| **Medication management: textual data on missed care** |  | 117 (12.0) |
| **Establishing a relationship with patients: textual data on missed care** |  | 181 (18.5) |
| **Talking and listening: textual data on missed care** |  | 97 (9.9) |
| **Non-verbal communication: textual data on missed care** |  | 173 (17.7) |
| **Shared decision-making: textual data on missed care** |  | 97 (9.9) |
| **Communicating with relatives, carers and significant others: textual data on missed care** |  | 116 (11.9) |
| **Dignity and respect: textual data on missed care** |  | 108 (11.0) |
| **Respecting patients’ values and beliefs: textual data on missed care** |  | 90 (9.2) |
| **Emotional wellbeing, anxiety and depression: textual data on missed care** |  | 90 (9.2) |
| **Quantitative barriers to hygiene, personal cleansing and toileting** | Lack of privacy for the patient | 304 |
|  | Wearing PPE | 261 |
|  | Severity of the patient’s condition | 230 |
|  | Frequent changes in hospital, Trust or organizational policies | 208 |
|  | Lack of personnel, skill mix, catering, housekeeping or dietetic support | 199 |
|  | Fear of catching COVID-19 | 180 |
|  | Difficulties taking items / equipment in and out of isolation rooms for patients nursed in these environments | 169 |
|  | Lack of knowledge about COVID-19 | 164 |
|  | Lack of appropriate PPE | 153 |
|  | Not enough physical resources such as equipment/washing facilities/stock items e.g. water jugs, disposable cups, patients’ teeth | 150 |
|  | Lack of ability to establish a meaningful rapport with the patient | 102 |
|  | Lack of access to changing facilities for PPE | 99 |
|  | Competing requirements of essential medical interventions | 76 |
|  | Lack of time | 74 |
|  | Lack of leadership from senior nurses or managers | 63 |
|  | Lack of personal psychological support | 61 |
|  | Lack of relevant personal expertise | 57 |
|  | Lack of information about the ward or patient | 56 |
|  | Lack of personal emotional capacity | 52 |
|  | Other | 27 |
|  | Lack of ability to regulate the environment (noise level, lighting, remote monitoring) | 0 |
|  | Total barriers selected | 595 |
| **Quantitative barriers to eating and drinking** | Difficulties taking items / equipment in and out of isolation rooms for patients nursed in these environments | 228 |
|  | Severity of the patient’s condition | 155 |
|  | Lack of personnel, skill mix, catering, housekeeping or dietetic support | 134 |
|  | Wearing PPE | 119 |
|  | Lack of time | 110 |
|  | Fear of catching COVID-19 | 104 |
|  | Not enough physical resources such as equipment/washing facilities/stock items e.g. water jugs, disposable cups, patients’ teeth | 99 |
|  | Frequent changes in hospital, Trust or organizational policies | 97 |
|  | Lack of knowledge about COVID-19 | 82 |
|  | Lack of appropriate PPE | 81 |
|  | Inability to meet the patient’s dietary requirements | 56 |
|  | Competing requirements of essential medical interventions | 53 |
|  | Lack of access to changing facilities for PPE | 39 |
|  | Lack of information about the ward or patient | 37 |
|  | Lack of ability to establish a meaningful rapport with the patient | 34 |
|  | Lack of leadership from senior nurses or managers | 32 |
|  | Lack of relevant personal expertise | 29 |
|  | Other | 25 |
|  | Lack of personal psychological support | 21 |
|  | Lack of personal emotional capacity | 13 |
|  | Total barriers selected | 421 |
| **Quantitative barriers to rest and sleep** | Severity of the patient’s condition | 164 |
|  | Lack of ability to regulate the environment (noise level, lighting, remote monitoring) | 160 |
|  | Difficulties taking items / equipment in and out of isolation rooms for patients nursed in these environments | 101 |
|  | Wearing PPE | 69 |
|  | Competing requirements of essential medical interventions | 65 |
|  | Lack of time | 48 |
|  | Frequent changes in hospital, Trust or organizational policies | 48 |
|  | Lack of knowledge about COVID-19 | 44 |
|  | Fear of catching COVID-19 | 43 |
|  | Not enough physical resources such as equipment/washing facilities/stock items e.g. water jugs, disposable cups, patients’ teeth | 40 |
|  | Lack of personnel, skill mix, catering, housekeeping or dietetic support | 36 |
|  | Lack of appropriate PPE | 36 |
|  | Lack of information about the ward or patient | 26 |
|  | Lack of ability to establish a meaningful rapport with the patient | 24 |
|  | Lack of personal psychological support | 21 |
|  | Other | 21 |
|  | Lack of access to changing facilities for PPE | 20 |
|  | Lack of relevant personal expertise | 19 |
|  | Lack of leadership from senior nurses or managers | 18 |
|  | Lack of personal emotional capacity | 13 |
|  | Total barriers selected | 329 |
| **Quantitative barriers to mobility** | Difficulties taking items / equipment in and out of isolation rooms for patients nursed in these environments | 176 |
|  | Severity of the patient’s condition | 171 |
|  | Lack of personnel, skill mix, catering, housekeeping or dietetic support | 124 |
|  | Lack of ability to regulate the environment (noise level, lighting, remote monitoring) | 124 |
|  | Wearing PPE | 103 |
|  | Lack of time | 101 |
|  | Not enough physical resources such as equipment/washing facilities/stock items e.g. water jugs, disposable cups, patients’ teeth | 98 |
|  | Fear of catching COVID-19 | 75 |
|  | Frequent changes in hospital, Trust or organizational policies | 60 |
|  | Lack of knowledge about COVID-19 | 59 |
|  | Competing requirements of essential medical interventions | 56 |
|  | Lack of appropriate PPE | 51 |
|  | Lack of relevant personal expertise | 33 |
|  | Lack of access to changing facilities for PPE | 28 |
|  | Other | 26 |
|  | Lack of leadership from senior nurses or managers | 22 |
|  | Lack of information about the ward or patient | 21 |
|  | Lack of personal psychological support | 21 |
|  | Lack of ability to establish a meaningful rapport with the patient | 20 |
|  | Lack of personal emotional capacity | 9 |
|  | Total barriers selected | 364 |
| **Quantitative barriers to patient comfort** | Severity of the patient’s condition | 158 |
|  | Difficulties taking items / equipment in and out of isolation rooms for patients nursed in these environments | 115 |
|  | Lack of knowledge about COVID-19 | 111 |
|  | Wearing PPE | 105 |
|  | Not enough physical resources such as equipment/washing facilities/stock items e.g. water jugs, disposable cups, patients’ teeth | 94 |
|  | Lack of personnel, skill mix, catering, housekeeping or dietetic support | 86 |
|  | Lack of time | 72 |
|  | Fear of catching COVID-19 | 69 |
|  | Frequent changes in hospital, Trust or organizational policies | 69 |
|  | Lack of appropriate PPE | 53 |
|  | Competing requirements of essential medical interventions | 43 |
|  | Lack of relevant personal expertise | 40 |
|  | Lack of ability to establish a meaningful rapport with the patient | 39 |
|  | Lack of personal psychological support | 28 |
|  | Lack of information about the ward or patient | 27 |
|  | Lack of access to changing facilities for PPE | 23 |
|  | Lack of personal emotional capacity | 19 |
|  | Lack of leadership from senior nurses or managers | 19 |
|  | Other | 18 |
|  | Total barriers selected | 330 |
| **Quantitative barriers to patient safety** | Wearing PPE | 119 |
|  | Lack of personnel, skill mix, catering, housekeeping or dietetic support | 114 |
|  | Severity of the patient’s condition | 112 |
|  | Difficulties taking items / equipment in and out of isolation rooms for patients nursed in these environments | 95 |
|  | Lack of time | 88 |
|  | Lack of knowledge about COVID-19 | 84 |
|  | Frequent changes in hospital, Trust or organizational policies | 81 |
|  | Not enough physical resources such as equipment/washing facilities/stock items e.g. water jugs, disposable cups, patients’ teeth | 66 |
|  | Fear of catching COVID-19 | 65 |
|  | Lack of appropriate PPE | 58 |
|  | Competing requirements of essential medical interventions | 52 |
|  | Lack of relevant personal expertise | 48 |
|  | Lack of information about the ward or patient | 43 |
|  | Lack of leadership from senior nurses or managers | 39 |
|  | Lack of ability to establish a meaningful rapport with the patient | 28 |
|  | Lack of personal psychological support | 27 |
|  | Lack of access to changing facilities for PPE | 27 |
|  | Lack of personal emotional capacity | 25 |
|  | Other | 11 |
|  | Total barriers selected | 301 |
| **Quantitative barriers to medication management** | Difficulties taking items / equipment in and out of isolation rooms for patients nursed in these environments | 84 |
|  | Lack of personnel, skill mix, catering, housekeeping or dietetic support | 84 |
|  | Severity of the patient’s condition | 79 |
|  | Wearing PPE | 73 |
|  | Not enough physical resources such as equipment/washing facilities/stock items e.g. water jugs, disposable cups, patients’ teeth | 64 |
|  | Lack of knowledge about COVID-19 | 61 |
|  | Lack of time | 60 |
|  | Frequent changes in hospital, Trust or organizational policies | 45 |
|  | Fear of catching COVID-19 | 38 |
|  | Competing requirements of essential medical interventions | 37 |
|  | Lack of relevant personal expertise | 36 |
|  | Lack of information about the ward or patient | 32 |
|  | Lack of appropriate PPE | 28 |
|  | Lack of leadership from senior nurses or managers | 25 |
|  | Lack of personal psychological support | 17 |
|  | Lack of ability to establish a meaningful rapport with the patient | 16 |
|  | Lack of personal emotional capacity | 15 |
|  | Lack of access to changing facilities for PPE | 15 |
|  | Other | 8 |
|  | Total barriers selected | 239 |
| **Quantitative barriers to establishing a relationship with patients** | Wearing PPE | 228 |
|  | Severity of the patient’s condition | 113 |
|  | Lack of time | 104 |
|  | Fear of catching COVID-19 | 86 |
|  | Lack of ability to establish a meaningful rapport with the patient | 76 |
|  | Difficulties taking items / equipment in and out of isolation rooms for patients nursed in these environments | 50 |
|  | Lack of knowledge about COVID-19 | 49 |
|  | Lack of personnel, skill mix, catering, housekeeping or dietetic support | 40 |
|  | Frequent changes in hospital, Trust or organizational policies | 39 |
|  | Lack of appropriate PPE | 37 |
|  | Lack of personal psychological support | 36 |
|  | Competing requirements of essential medical interventions | 36 |
|  | Lack of personal emotional capacity | 33 |
|  | Lack of information about the ward or patient | 32 |
|  | Not enough physical resources such as equipment/washing facilities/stock items e.g. water jugs, disposable cups, patients’ teeth | 24 |
|  | Lack of relevant personal expertise | 21 |
|  | Lack of access to changing facilities for PPE | 19 |
|  | Lack of leadership from senior nurses or managers | 18 |
|  | Other | 7 |
|  | Total barriers selected | 346 |
| **Quantitative barriers to talking and listening** | Wearing PPE | 227 |
|  | Lack of time | 95 |
|  | Severity of the patient’s condition | 85 |
|  | Fear of catching COVID-19 | 73 |
|  | Lack of ability to establish a meaningful rapport with the patient | 56 |
|  | Lack of knowledge about COVID-19 | 46 |
|  | Difficulties taking items / equipment in and out of isolation rooms for patients nursed in these environments | 40 |
|  | Lack of personnel, skill mix, catering, housekeeping or dietetic support | 35 |
|  | Lack of appropriate PPE | 31 |
|  | Lack of personal psychological support | 30 |
|  | Frequent changes in hospital, Trust or organizational policies | 30 |
|  | Lack of personal emotional capacity | 26 |
|  | Competing requirements of essential medical interventions | 26 |
|  | Lack of information about the ward or patient | 25 |
|  | Not enough physical resources such as equipment/washing facilities/stock items e.g. water jugs, disposable cups, patients’ teeth | 19 |
|  | Lack of leadership from senior nurses or managers | 17 |
|  | Lack of access to changing facilities for PPE | 16 |
|  | Lack of relevant personal expertise | 15 |
|  | Other | 11 |
|  | Total barriers selected | 324 |
| **Quantitative barriers to non-verbal communication** | Wearing PPE | 230 |
|  | Lack of time | 72 |
|  | Severity of the patient’s condition | 62 |
|  | Fear of catching COVID-19 | 50 |
|  | Lack of ability to establish a meaningful rapport with the patient | 35 |
|  | Difficulties taking items / equipment in and out of isolation rooms for patients nursed in these environments | 27 |
|  | Lack of personnel, skill mix, catering, housekeeping or dietetic support | 26 |
|  | Lack of knowledge about COVID-19 | 25 |
|  | Lack of appropriate PPE | 23 |
|  | Frequent changes in hospital, Trust or organizational policies | 22 |
|  | Lack of information about the ward or patient | 21 |
|  | Not enough physical resources such as equipment/washing facilities/stock items e.g. water jugs, disposable cups, patients’ teeth | 19 |
|  | Lack of personal psychological support | 16 |
|  | Lack of personal emotional capacity | 14 |
|  | Competing requirements of essential medical interventions | 13 |
|  | Lack of relevant personal expertise | 11 |
|  | Lack of access to changing facilities for PPE | 9 |
|  | Lack of leadership from senior nurses or managers | 8 |
|  | Other | 5 |
|  | Total barriers selected | 298 |
| **Quantitative barriers to shared decision-making** | Severity of the patient’s condition | 84 |
|  | Wearing PPE | 81 |
|  | Lack of time | 52 |
|  | Lack of knowledge about COVID-19 | 49 |
|  | Fear of catching COVID-19 | 33 |
|  | Lack of personnel, skill mix, catering, housekeeping or dietetic support | 30 |
|  | Lack of ability to establish a meaningful rapport with the patient | 27 |
|  | Frequent changes in hospital, Trust or organizational policies | 25 |
|  | Difficulties taking items / equipment in and out of isolation rooms for patients nursed in these environments | 20 |
|  | Lack of information about the ward or patient | 20 |
|  | Lack of relevant personal expertise | 19 |
|  | Competing requirements of essential medical interventions | 18 |
|  | Lack of leadership from senior nurses or managers | 16 |
|  | Lack of personal emotional capacity | 11 |
|  | Lack of appropriate PPE | 10 |
|  | Lack of personal psychological support | 9 |
|  | Not enough physical resources such as equipment/washing facilities/stock items e.g. water jugs, disposable cups, patients’ teeth | 8 |
|  | Lack of access to changing facilities for PPE | 6 |
|  | Other | 3 |
|  | Total barriers selected | 178 |
| **Quantitative barriers to communicating with relatives, carers and significant others** | Lack of time | 108 |
|  | Wearing PPE | 99 |
|  | Not enough physical resources such as equipment/washing facilities/stock items e.g. water jugs, disposable cups, patients’ teeth | 91 |
|  | Severity of the patient’s condition | 66 |
|  | Frequent changes in hospital, Trust or organizational policies | 54 |
|  | Difficulties taking items / equipment in and out of isolation rooms for patients nursed in these environments | 47 |
|  | Other | 46 |
|  | Lack of knowledge about COVID-19 | 41 |
|  | Lack of ability to establish a meaningful rapport with the patient | 31 |
|  | Fear of catching COVID-19 | 29 |
|  | Competing requirements of essential medical interventions | 28 |
|  | Lack of personnel, skill mix, catering, housekeeping or dietetic support | 27 |
|  | Lack of information about the ward or patient | 25 |
|  | Lack of relevant personal expertise | 21 |
|  | Lack of leadership from senior nurses or managers | 17 |
|  | Lack of appropriate PPE | 14 |
|  | Lack of personal emotional capacity | 13 |
|  | Lack of personal psychological support | 12 |
|  | Lack of access to changing facilities for PPE | 6 |
|  | Total barriers selected | 265 |
| **Quantitative barriers to**  **dignity and respect** | Wearing PPE | 84 |
|  | Severity of the patient’s condition | 70 |
|  | Lack of time | 68 |
|  | Lack of personnel, skill mix, catering, housekeeping or dietetic support | 49 |
|  | Lack of knowledge about COVID-19 | 40 |
|  | Fear of catching COVID-19 | 40 |
|  | Difficulties taking items / equipment in and out of isolation rooms for patients nursed in these environments | 38 |
|  | Not enough physical resources such as equipment/washing facilities/stock items e.g. water jugs, disposable cups, patients’ teeth | 34 |
|  | Frequent changes in hospital, Trust or organizational policies | 27 |
|  | Competing requirements of essential medical interventions | 24 |
|  | Lack of information about the ward or patient | 22 |
|  | Lack of ability to establish a meaningful rapport with the patient | 19 |
|  | Lack of personal psychological support | 18 |
|  | Lack of appropriate PPE | 18 |
|  | Lack of leadership from senior nurses or managers | 17 |
|  | Lack of relevant personal expertise | 16 |
|  | Lack of personal emotional capacity | 13 |
|  | Lack of access to changing facilities for PPE | 12 |
|  | Other | 10 |
|  | Total barriers selected | 179 |
| **Quantitative barriers to respecting values and beliefs** | Wearing PPE | 55 |
|  | Severity of the patient’s condition | 53 |
|  | Lack of time | 45 |
|  | Lack of information about the ward or patient | 36 |
|  | Fear of catching COVID-19 | 34 |
|  | Lack of personnel, skill mix, catering, housekeeping or dietetic support | 31 |
|  | Lack of knowledge about COVID-19 | 28 |
|  | Difficulties taking items / equipment in and out of isolation rooms for patients nursed in these environments | 23 |
|  | Frequent changes in hospital, Trust or organizational policies | 22 |
|  | Lack of ability to establish a meaningful rapport with the patient | 21 |
|  | Not enough physical resources such as equipment/washing facilities/stock items e.g. water jugs, disposable cups, patients’ teeth | 20 |
|  | Lack of relevant personal expertise | 17 |
|  | Competing requirements of essential medical interventions | 15 |
|  | Lack of appropriate PPE | 15 |
|  | Other | 14 |
|  | Lack of personal emotional capacity | 11 |
|  | Lack of personal psychological support | 11 |
|  | Lack of leadership from senior nurses or managers | 10 |
|  | Lack of access to changing facilities for PPE | 6 |
|  | Total barriers selected | 152 |
| **Quantitative barriers to wellbeing, depression and anxiety** | Wearing PPE | 111 |
|  | Lack of time | 93 |
|  | Severity of the patient’s condition | 83 |
|  | Lack of knowledge about COVID-19 | 71 |
|  | Fear of catching COVID-19 | 56 |
|  | Lack of personnel, skill mix, catering, housekeeping or dietetic support | 53 |
|  | Lack of ability to establish a meaningful rapport with the patient | 44 |
|  | Lack of personal psychological support | 39 |
|  | Frequent changes in hospital, Trust or organizational policies | 33 |
|  | Lack of personal emotional capacity | 32 |
|  | Difficulties taking items / equipment in and out of isolation rooms for patients nursed in these environments | 31 |
|  | Lack of information about the ward or patient | 31 |
|  | Lack of relevant personal expertise | 28 |
|  | Competing requirements of essential medical interventions | 26 |
|  | Not enough physical resources such as equipment/washing facilities/stock items e.g. water jugs, disposable cups, patients’ teeth | 23 |
|  | Lack of appropriate PPE | 23 |
|  | Lack of leadership from senior nurses or managers | 18 |
|  | Other | 17 |
|  | Lack of access to changing facilities for PPE | 12 |
|  | Total barriers selected | 233 |
| **Hygiene, personal cleansing and toileting: textual data on barriers** |  | 249 (25.5) |
| **Eating and drinking: textual data on barriers** |  | 189 (19.3) |
| **Rest and sleep: textual data on barriers** |  | 148 (15.1) |
| **Mobility: textual barriers to mobility** |  | 166 (17.0) |
| **Patient comfort: textual data on barriers** |  | 134 (13.7) |
| **Patient safety: textual data on barriers** |  | 109 (11.1) |
| **Medication management: textual data on barriers** |  | 88 (9.0) |
| **Establishing a relationship with patients: textual data on barriers** |  | 123 (12.6) |
| **Talking and listening: textual data on barriers** |  | 122 (12.5) |
| **Non-verbal communication: textual data on barriers** |  | 89 (9.1) |
| **Shared decision-making: textual data on barriers** |  | 68 (7.0) |
| **Communicating with relatives, carers and significant others: textual data on barriers** |  | 109 (11.1) |
| **Dignity and respect: textual data on barriers** |  | 73 (7.5) |
| **Respecting patients’ values and beliefs: textual data on barriers** |  | 51 (5.2) |
| **Emotional wellbeing, anxiety and depression: textual data on barriers** |  | 78 (8.0) |
